# Relationship between indices of circulating blood cells and bone homeostasis in osteoporosis

**DOI:** 10.3389/fendo.2022.965290

**Published:** 2022-09-05

**Authors:** Yuan Li, Weimin Hao, Jianming Guan, Bo Li, Li Meng, Shuangjiao Sun, Tianyuan Sheng, Shuangxi Dong, Qian Zhou, Mingjie Liu, Zhongkai Zhang, Tao Shen, Yuemao Shen, Baobing Zhao

**Affiliations:** ^1^ Key Lab of Chemical Biology, Ministry of Education (MOE), School of Pharmaceutical Sciences, Cheeloo College of Medicine, Shandong University, Jinan, China; ^2^ Suzhou Research Institute, Shandong University, Suzhou, China; ^3^ National Medical Products Administration (NMPA) Key Laboratory for Technology Research and Evaluation of Drug Products, School of Pharmaceutical Sciences, Cheeloo College of Medicine, Shandong University, Jinan, China; ^4^ Department of Spine Surgery, Heze Municipal Hospital, Heze, Shandong, China; ^5^ Department of Hematology, Heze Municipal Hospital, Heze, Shandong, China; ^6^ Department of Orthopedics, Sun Yat-Sen Memorial Hospital of Sun Yat-Sen University, Guangzhou, China; ^7^ Department of Orthopedics, Shandong Provincial Hospital Affiliated to Shandong First Medical University, Jinan, China

**Keywords:** osteoporosis, hematopoiesis, bone homeostasis, circulating blood cells, bone mineral density

## Abstract

Bone development have been shown to play an important role in regulating hematopoiesis as one major component of bone marrow microenvironment. Recent studies support the notion that there is an intricate relationship between hematopoiesis and bone homeostasis, however, little is known about the alterations in the hematopoietic lineages in pathologic conditions. Using various osteoporotic mouse models, we show here that bone microarchitecture abnormalities alter parameters of peripheral blood cells. The level of white blood cells is dynamics and negatively correlated with bone mineral density during the progression of osteoporosis. Furthermore, our clinical data confirm that osteoporosis is associated with abnormal circulating blood cell counts. These results demonstrated a causal link that osteoporosis is accompanied by the altered circulating blood cells, supporting the idea of a close interplay between hematopoiesis and bone homeostasis. Our study would propose that routine complete blood count might be applied as a potential diagnostic and putative marker for osteoporosis.

## Introduction

Osteoporosis (OP) is a type of systemic skeletal and age-related disease characterized by stumpy bone mass and the microarchitectural weakening, leading to elevated bone fragility and thus a subsequent increased risk of fractures (1–3). OP frequently occurs in postmenopausal women, affecting about 1 out of 3 women over 50 years old (4, 5). Notably, nearly half of women have experienced fractures one time after 50 years old (6–8). With the aging of the population, the incidence of osteoporotic fractures might be increased by approximately 25% in the next 10 years. Loss of bone mineral density (BMD) is the major diagnostic metrics of OP due to an imbalance between osteoclast-mediated bone resorption and osteoblast-mediated bone formation in these patients (9–11). However, this diagnostic method is usually performed after the onset of the symptoms of osteoporotic patients, which might delay all the efforts at prevention and treatment.

Recent studies support the notion that there is an intricate relationship between hematopoiesis and bone homeostasis in normal steady states (12, 13). Bone marrow is responsible for providing an appropriate and specific microenvironment for the maintenance of bone homeostasis and blood cells formation, in which different stages of hematopoiesis, bone formation, bone resorption and variety of immune responses are precisely controlled (14–17). The osteogenic niche comprised of osteoprogenitors, preosteoblasts, osteoblasts, osteocytes and osteoclasts, exerts established functions in providing unique niches and anatomical spaces for supporting normal hematopoiesis (18, 19). On the other hand, hematopoiesis plays a critical role in the precise regulation of special microenvironment which are directly related with the bone and blood physiology (20–22).

Given this, the nature of the relationships between blood cells formation and bone homeostasis remains undefined outside of normal physiological states. In this study, we explored the relevance of these relationships to osteoporotic conditions. Our findings demonstrated a causal link that osteoporosis is accompanied by the altered circulating blood cells, supporting the idea of a close interplay between hematopoiesis and bone homeostasis. Our study would propose that routine complete blood count is applied as a potential diagnostic and putative marker for osteoporosis. A better understanding of these relationships can offer novel insights into routine assessment and early diagnosis for bone loss related diseases including osteoporosis.

## Material and methods

### Ethical statement

This study was approved by the Ethical Committee of Heze municipal Hospital. The animal use protocols in this study were conducted in accordance with the Medical Ethical Committee (MEC) and approved by the Institutional Animal Care and Use Committees at Shandong University.

### Study participants

Between January, 2020 and October, 2021, 608 osteoporotic patients who were received treatment at Heze municipal Hospital included in this study. All patients were diagnosed with osteoporosis according to the 2015 Guidelines for the Diagnosis and Treatment of Osteoporosis issued by the Branch of Osteoporosis and Bone Mineral Salt Diseases, Chinese Medical Association. Besides, 527 healthy participants, who were referred to Heze municipal Hospital for routine checkups, were recruited in this study.

The following exclusion criteria were employed: (1) do not meet the diagnosis of osteoporosis; (2) Rheumatoid arthritis, diabetes mellitus, hyperthyroidism, and other secondary osteoporosis; (3) history of anemia or recent blood donation in the past 2 months; (4) history of serious cardiovascular and cerebrovascular diseases; (5) history of malignancy tumor, autoimmune disease, hematological disorders, renal and liver failure, liver cirrhosis, thyroid or parathyroid disorders and current infection.

### Data collection

The general information and hematological indices, including sex, age, white blood cells (WBC), number of neutrophils (NEUT#), number of lymphocytes (LYMPH#), number of monocytes (MONO#), number of eosinophils (EO#), number of basophils (BASO#), red blood cells (RBC), hemoglobin (HGB), hematocrit (HCT), mean corpuscular volume (MCV), mean corpuscular hemoglobin (MCH), red cell distribution width-CV (RDW-CV), platelets (PLT), platelets and platelet distribution width (PDW), mean platelet volume (MPV), platelet hematocrit (PCT), were recorded and analyzed.

### Construction of OVX-related osteoporotic mouse models

Sixty-four female C57BL/6J mice (about 8 weeks old, 18 to 20 g) were purchased from Beijing Vital River Laboratory Animal Technology Co., Ltd. The mice were housed under standard laboratory conditions with free access to sterile standard mouse chow and water. The condition was at room temperature (22 ± 2°C) with a relative humidity (55 ± 5%) under a normal 12 h light/12 h dark cycle. After 1 week of acclimation to the environment, all the mice were randomly assigned to two groups: sham group (n=32) and OVX group (n=32).

The surgical ovariectomy (OVX) operation was conducted according to the previous study (23). The bilateral ovaries were gently removed from mice in OVX group. while some adipose tissue around the ovaries were removed from mice in sham group. After eight weeks, the BMD of mice was detected to evaluate the establishment of osteoporotic mouse models and the relationship between BMD and blood cell counts of OVX-related osteoporotic mouse models was analyzed at this timepoint.

### Measurement of hematological indices in mice

For the duration of this study, the blood samples of mice were collected from the caudal vena cava for analyzing the hematological indices in mice. The hematological indices, white blood cells (WBC), lymphocytes (LYMPH#), monocytes (MONO#), neutrophils (NEUT#), red blood cells (RBC), hematocrit (HCT), hemoglobin (HGB), red cell distribution width (RDW), mean platelet volume (MPV), platelets (PLT) and platelet distribution width (PDW) were measured using Hematology Analyzer BC-2800VET (Mindray, China). The hematology analyzer has been routinely checked to guarantee that the hematological parameters of blood samples are within the precision specifications.

### Detection of BMD in mice

The whole-body BMD of mice was measured by using XR-600 digital fast dual-energy X-ray scanning absorptiometer (NORLAND, USA) in Bone Density Measuring Instrument Sharing Platform of Shandong University and Health analysis and test center of Shandong university. The mice from different groups were placed on a specimen tray and kept them ventral side down with each limb and tail maintained away from the body. The full-body scans and data were obtained by the dual-energy X-ray scanning absorptiometer and manufacturer supplied software.

### Establishment of iron overload-induced osteoporotic mouse models

Twenty-four male C57BL/6J mice (about 8 weeks old, 18 to 20 g) were purchased from Beijing Vital River Laboratory Animal Technology Co., Ltd. The mice were housed in the condition mentioned above. After 1 week of acclimation to the environment, all the mice were randomly divided to two groups: control (CTL) group (n=12) and iron overload-induced osteoporosis (OP) group (n=12). The mice from OP group were injected with 0.016 mL/g of iron dextran (diluted by normal saline to 10 mg/mL) every other day for eight weeks. The mice from CTL group were injected with 0.016 mL/g of normal saline by intraperitoneal injection every other day for eight weeks. After eight weeks, the BMD of mice was examined to ensure the construction of osteoporotic mouse models and the relationship between BMD and blood cell counts of iron overload-induced osteoporotic mouse models was analyzed at this timepoint.

### Statistical analysis

Data were presented as mean values with standard deviations (mean ± SD). Data were double recorded and validated by using Excel spreadsheets before analysis was performed using Graphpad Prism 8 software. Data were validated for inconsistency, missing data and outliers to ensure their accuracy and quality. Correlation analysis between circulating blood cell counts and BMD was performed using Pearson correlation and multivariate linear regression analysis. *P*<0.05 indicated that the difference was considered statistically significant.

## Results

### Comparison of the hematological indices of osteoporotic patients and healthy controls

To further analyze the hematological indices in osteoporotic patients, we collected the general information and hematological indices of clinical osteoporotic patients and healthy controls. In this study, the information of 608 osteoporosis patients and 527 healthy control subjects were collected, and the baseline characteristics of the study participants were shown in **Table 1**. Among these participants in the study, the males respectively accounted for19.23% in control group and 18.98% in osteoporotic group, and there was no difference (*p*>0.05). Besides, the mean age was 71.56 ± 8.90 years and 71.23 ± 8.57 years in control group and osteoporotic group, respectively. And the results showed that there was no difference in age between control group and osteoporotic group (*p*>0.05).

**Table 1:** Characteristics and hematological indices of osteoporotic patients.

Characteristics	Controls (n=527)	Patients (n=608)	*P* values
Age (years)	71.56 ± 8.90	71.23 ± 8.57	>0.05
Sex (male/female)	19.23% (85/442)	18.98% (97/511)	>0.05
WBC	5.86 ± 1.59	6.34 ± 2.02	<0.001
NEUT#	3.59 ± 1.39	4.12 ± 1.83	<0.001
LYMPH#	1.70 ± 0.55	1.64 ± 0.64	>0.05
MONO#	0.37 ± 0.12	0.42 ± 0.17	<0.001
EO#	0.12 ± 0.11	0.12 ± 0.10	>0.05
BASO#	0.024 ± 0.017	0.027 ± 0.015	>0.05
RBC	4.12 ± 0.42	4.06 ± 0.49	>0.05
HGB	126.8 ± 12.98	125.1 ± 19.29	>0.05
HCT	38.55 ± 13.47	37.93 ± 12.81	>0.05
MCV	92.22 ± 5.92	92.53 ± 6.11	>0.05
MCH	30.84 ± 1.72	30.77 ± 2.08	>0.05
RDW-CV	13.35 ± 12.41	13.43 ± 11.58	>0.05
PLT	224.88 ± 63.57	227.89 ± 69.77	>0.05
PDW	13.30 ± 9.05	12.89 ± 8.58	>0.05
MPV	10.86 ± 1.17	10.32 ± 1.10	>0.05
PCT	0.24 ± 0.06	0.23 ± 0.06	>0.05

WBC, white blood cells; NEUT#, number of neutrophils; LYMPH#, number of lymphocytes; MONO#, number of monocytes; EO#, number of eosinophils; BASO#, number of basophils; RBC, red blood cells; HGB, hemoglobin; HCT, hematocrit; MCV, mean corpuscular volume; MCH, mean corpuscular hemoglobin; RDW-CV, red cell distribution width-CV; PLT, platelets; PDW, platelets and platelet distribution width; MPV, mean platelet volume; PCT, platelet hematocrit.

Next, the hematological indices in osteoporotic patients and control group were compared. The results showed that the counts of WBC, NEUT# and MONO# were much higher in osteoporotic group than that in control group (**Table 1**). The variables of WBC, NEUT# and MONO# between these two groups were significant (*p*<0.001 respectively). And the results of comparison of LYMPH#, EO#, BASO#, RBC, HGB, HCT, MCV, MCH, RDW-CV, PLT, PDW, MPV, PCT showed that there was no statistical difference in these hematological indices between control group and osteoporotic group (*p*>0.05).

To investigate whether the alteration of hematological indices in osteoporotic patients is related to the estrogen deficiency-related menopause, we further analyzed the hematological indices in only male osteoporotic patients. The results showed that the counts of WBC, NEUT# and MONO# were much higher in male osteoporotic patients than that in male controls (*p*=0.0394, 0.0019 and 0.0062 respectively) (**Table 2**). Other hematological indices showed no statistical difference between the two group (*p *>0.05). These findings indicated that the increased counts of white blood cells, neutrophils and monocytes might be used as a novel prognostic factor for osteoporosis.

**Table 2:** Comparison of hematological indices between osteoporotic patients and healthy controls (only male).

Characteristics	Controls (n=85)	Patients (n=97)	*P* values
WBC	6.18 ± 1.50	6.69 ± 1.80	<0.001
NEUT#	3.87 ± 1.25	4.53 ± 1.53	<0.001
LYMPH#	1.66 ± 0.60	1.51 ± 0.58	>0.05
MONO#	0.43 ± 0.11	0.49 ± 0.18	<0.001
EO#	0.16 ± 0.16	0.15 ± 0.16	>0.05
BASO#	0.022 ± 0.016	0.03 ± 0.01	>0.05
RBC	4.30 ± 0.39	4.17 ± 0.53	>0.05
HGB	135.4 ± 10.77	134 ± 11.03	>0.05
HCT	39.60 ± 3.07	38.48 ± 5.41	>0.05
MCV	93.51 ± 4.56	93.13 ± 5.85	>0.05
MCH	31.55 ± 1.72	31.39 ± 2.31	>0.05
RDW-CV	12.80 ± 0.64	12.91 ± 1.53	>0.05
PLT	199.74 ± 51.10	209.67 ± 52.03	>0.05
PDW	12.86 ± 2.01	12.15 ± 2.63	>0.05
MPV	10.86 ± 1.00	10.04 ± 0.88	>0.05
PCT	0.22 ± 0.05	0.21 ± 0.05	>0.05

WBC, white blood cells; NEUT#, number of neutrophils; LYMPH#, number of lymphocytes; MONO#, number of monocytes; EO#, number of eosinophils; BASO#, number of basophils; RBC, red blood cells; HGB, hemoglobin; HCT, hematocrit; MCV, mean corpuscular volume; MCH, mean corpuscular hemoglobin; RDW-CV, red cell distribution width-CV; PLT, platelets; PDW, platelets and platelet distribution width; MPV, mean platelet volume; PCT, platelet hematocrit.

### Construction and characterization of osteoporotic mice

**Figure 1:**
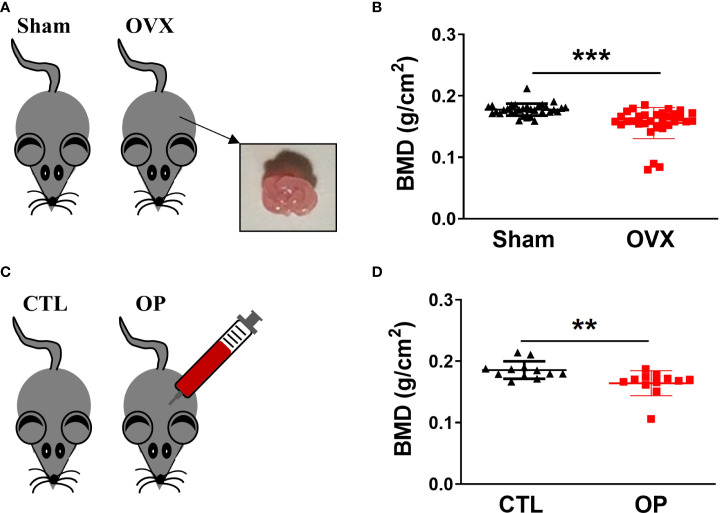
Construction of osteoporotic mice and identification of BMD. **(A)** Estrogen deficiency-related osteoporotic mouse models were constructed *via* bilateral ovariectomy (OVX) operation. **(B)** The whole body of BMD in estrogen deficiency-related osteoporotic mice was measured using dual energy-ray absorptiometry. n = 32. **(C)** Iron overload-induced osteoporotic mouse models were established *via* intraperitoneal injection of iron dextran. **(D)** Measurement of BMD in iron overload-induced osteoporotic mice. n=12. **p <0.01, ***p <0.001 versus sham or CTL. Sham, sham operation group; OVX, ovariectomy operation group; CTL, control group; OP, overload-induced osteoporotic group. BMD, bone mineral density.

To gain insight into the relationship between hematopoiesis and bone homeostasis in osteoporotic condition, two types of osteoporotic mice, including estrogen deficiency-related osteoporotic mouse models and iron overload-induced osteoporotic mouse models, were used for analysis. The estrogen deficiency-related osteoporotic mice were constructed by using bilateral ovariectomy (OVX) operation (**Figure 1A**). After 8 weeks, as the most clinically relevant factor, the whole-body BMD of mice was measured by using dual-energy X-ray scanning absorptiometer to quantify the osteoporotic changes. As anticipated, compared with mice from the sham group (n=32), the ovariectomized mice (n=32) showed a significant reduction in BMD, which was regarded as typical osteoporotic alterations (**Figure 1B**). Besides, iron overload-induced osteoporotic mice were obtained *via* intraperitoneal injection of iron dextran (**Figure 1C**). Then, the BMD of mice was detected to evaluate the establishment of osteoporotic mouse models. Similarly, compared to the control group (n=12), the mice from overload-induced osteoporotic mice (n=12) demonstrated an obvious reduction in BMD (**Figure 1D**). These results confirmed that the osteoporotic mice were successfully established and the osteoporotic mice displayed the obvious reduction in BMD.

### Measurement of hematological indices in estrogen deficiency-related osteoporotic mice

**Figure 2:**
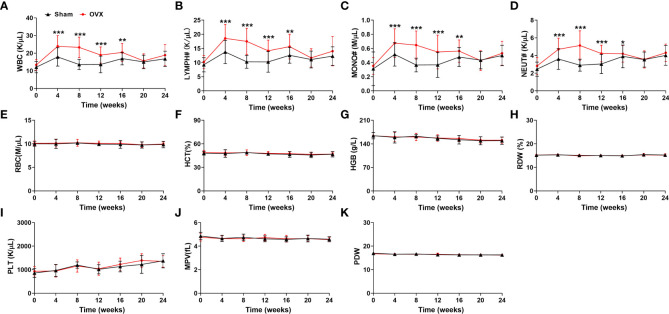
Analysis of hematological indices in estrogen deficiency-related osteoporotic mice. **(A–K)** The counts of WBC **(A)**, LYMPH# **(B)**, MONO# **(C)**, NEUT# **(D)**, RBC **(E)**, HCT **(F)**, HGB **(G)**, RDW **(H)**, PLT **(I)**, MPV **(J)** and PDW **(K)** in deficiency-related osteoporotic mice were examined by Hematology Analyzer. n = 32. *p <0.05, **p <0.01, ***p <0.001 versus sham. Sham, sham operation group; OVX, ovariectomy operation group; WBC, white blood cells; LYMPH#, lymphocytes; MONO#, monocytes; NEUT#, neutrophils; RBC, red blood cells; HCT, hematocrit; HGB, hemoglobin; RDW, red cell distribution width; PLT, platelets; MPV, mean platelet volume; PDW, platelet distribution width.

According to the recent studies, there is a closed link between osteoporosis and hematopoiesis (24–26). However, the hematological indices in osteoporotic conditions have not been fully explored. Thus, the blood samples of mice were collected from the caudal vena cava and the hematological indices were measured every four weeks. Among the hematological indices, the numbers of WBC, MONO#, LYMPH# and NEUT# in peripheral blood were much higher at 4, 8, 12, 16 weeks after OVX surgery in the OVX-related osteoporotic mice than the sham group (**Figures 2A–D**). And the elevated numbers of WBC, MONO#, LYMPH# and NEUT# in peripheral blood of osteoporotic mice were almost restored after 20 weeks of bilateral ovarian resection (**Figures 2A–D**). However, compared with mice in the sham group, there was no significant difference in the RBC, HCT, HGB, RDW, PLT, MPV and PDW of OVX-related osteoporotic mice (**Figures 2E–K**). These results suggested that WBC, MONO#, LYMPH# and NEUT# in peripheral blood might be correlated with the development of deficiency-related osteoporotic mice.

### Measurement of hematological indices in iron overload-induced osteoporotic mice

**Figure 3:**
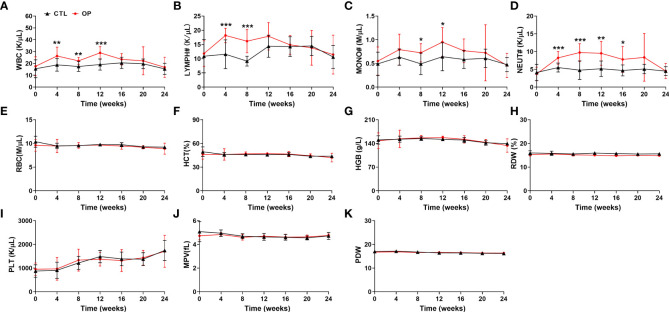
Detection of hematological indices in iron overload-induced osteoporotic mice. **(A–K)** The counts of WBC **(A)**, LYMPH# **(B)**, MONO# **(C)**, NEUT# **(D)**, RBC **(E)**, HCT **(F)**, HGB **(G)**, RDW **(H)**, PLT **(I)**, MPV **(J)** and PDW **(K)** were examined in iron overload-induced osteoporotic mice. n=12. *p <0.05, **p <0.01, ***p <0.001 versus CTL. CTL, control group; OP, overload-induced osteoporotic group; WBC, white blood cells; LYMPH#, lymphocytes; MONO#, monocytes; NEUT#, neutrophils; RBC, red blood cells; HCT, hematocrit; HGB, hemoglobin; RDW, red cell distribution width; PLT, platelets; MPV, mean platelet volume; PDW, platelet distribution width.

To present a comprehensive investigation of the potential relationship between osteoporosis and hematopoiesis, we then detected the hematological indices in iron overload-induced osteoporotic mice every four weeks. As show in **Figures 3A–D**, compared with mice from CTL group, the numbers of WBC, MONO#, LYMPH# and NEUT# in peripheral blood were significantly elevated in the iron overload-induced osteoporotic mice (**Figures 3A–D**). The increased numbers of WBC, MONO#, LYMPH# and NEUT# in peripheral blood were gradually retrieved after approximately 16 weeks (**Figures 3A–D**). Similarly, there was no obvious alteration in the RBC, HCT, HGB, RDW, PLT, MPV and PDW between CTL group and OP group (**Figures 3E–K**). These findings confirmed that there was close correlation between WBC, MONO#, LYMPH# and NEUT# in peripheral blood and osteoporosis.

### Correlation of peripheral blood cell counts with BMD in osteoporosis

**Figure 4:**
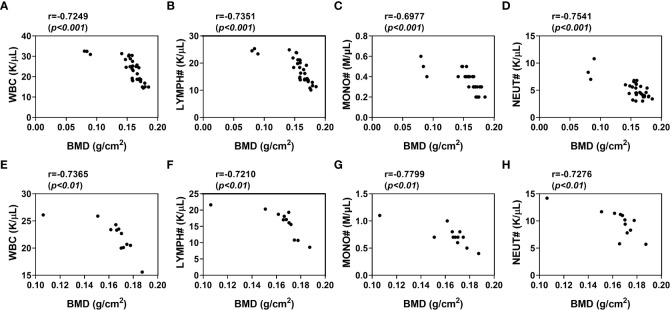
Correlation of WBC, lymphocytes, monocytes, neutrophils levels with BMD in osteoporotic mice after 8 weeks of osteoporotic mouse model construction. **(A–D)** Analysis of the correlation between WBC **(A)**, LYMPH# **(B)**, MONO# **(C)**, NEUT# **(D)** and BMD in estrogen deficiency-related osteoporotic mice **(E, F)** Analysis of the correlation between WBC **(E)**, LYMPH# **(F)**, MONO# **(G)**, NEUT# **(H)** and BMD in iron overload-induced osteoporotic mice. Sham, sham operation group; OVX, ovariectomy operation group; CTL, control group; OP, overload-induced osteoporotic group. BMD, bone mineral density; WBC, white blood cells; LYMPH#, lymphocytes; MONO#, monocytes; NEUT#, neutrophils.

The peripheral blood cell counts were altered in osteoporosis, which suggested that there might be association between peripheral blood cell counts and BMD. To verify this hypothesis, we analyzed the correlation between BMD and the hematological indices in the osteoporotic condition respectively after 8 weeks of osteoporotic mouse model construction. The peripheral blood cell counts were negatively correlated with BMD in estrogen deficiency-related osteoporosis, including WBC (*p *<0.001, r=-0.7249), MONO# (*p *<0.001, r=-0.7351), LYMPH# (*p *<0.001, r=-0.6977) and NEUT# (*p *<0.001, r=-0.7541) (**Figures 4A–D**).

To further comform the correlation between blood cell counts and BMD in osteoporosis, we also analyzed this correlation in iron overload-induced osteoporotic mice. As expected, there were significant associations between peripheral blood cell counts and BMD in these osteoporotic mice (**Figures 4E–H**). The BMD of iron overload-induced osteoporotic mice was respectively and negatively associated with WBC (*p *<0.01, r=-0.7365), MONO# (*p *<0.01, r=-0.7210), LYMPH# (*p *<0.01, r=-0.7799) and NEUT# (*p *<0.01, r=-0.7276). These data indicated that peripheral blood WBC, monocytes, lymphocytes and neutrophils counts were independently and negatively associated with BMD in osteoporosis.

## Discussion

Although OP is a chronic and age-dependent bone disease, its complications seriously affect the quality of life of osteoporotic patients and bring huge economic burden to the society (27, 28). With the increased lifespan of the human population, a higher proportion of the global population will be susceptible to osteoporosis (29–31). It is the current challenge to develop novel strategies to identify the high risk of osteoporosis so that the patients receive the corresponding treatment as early as possible (32–34). In this study, we demonstrated white blood cells level is dynamics and negatively correlated with bone mineral density during the progression of osteoporosis, supporting that routine complete blood count is applied as a potential diagnostic and putative marker for osteoporosis.

Accumulating evidences have indicated a close relationship between bone marrow hematopoiesis and bone formation (35, 36). There have also been some studies of the relationship between osteoporosis and indices of circulating blood cells. A study of Chinese postmenopausal women showed that the counts of RBC and HGB levels were much higher in osteoporosis compared with non-osteoporotic patients (37). While, in another study, the blood cell counts (including WBC, RBC and platelets) were significantly reduced in postmenopausal women from Seoul, Korea (38). On the contrary, our results indicated that WBC counts were increased in the peripheral blood from osteoporotic patients and osteoporotic mice, but there is no difference on the other indices of blood cells between osteoporosis and controls.

We also demonstrated that the counts of WBC were dynamics and negatively correlated with BMD in osteoporotic mice, which is consistent with a previous study in the postmenopausal osteoporosis (37). However, two independent investigations have also shown positive correlations or undetectable correlations between blood cell counts and BMD respectively (38, 39). These disparate findings may be due to the distinct comparations. There are many factors accounting for the inconsistent results from patients, including region, race, age and sex osteoporotic patients and controls. Indeed, our clinical data were collected from 97 male and 511 female osteoporotic patients, while the previous studies were mainly based on postmenopausal osteoporosis (37, 40). Furthermore, the hematological indices in osteoporotic patients and controls were separately determined in men, which showed that the counts of WBC, NEUT# and MONO# were also much higher in osteoporotic group than that in control group (**Table 2**). These results suggested that the alteration of hematological indices in osteoporotic patients was not limited to the estrogen deficiency-related menopause.

White blood cells-to-lymphocyte ratio have been reported to be independent predictors in many diseases including osteoporosis (41–44). In this study, we also calculated the rations of neutrophil to lymphocyte (NLR), platelet to lymphocyte (PLR) and monocyte to lymphocyte (MLR) with all the colleced data. The levels of NLR, PLR, and MLR were all higher than those in healthy control subjects (data not shown), which is consistent with the previous study (42). Thees findings provied further evidence to surpport that the value of NLR, PLR, and MLR might be used as novel potential predictors of osteoporosis.

The negtive correlation of bone mineral density and peripheral white blood cells indicated that the altered circulating white blood cells play roles in the progression of osteoporosis. The WBC subpopulations were immediately increased after 4 weeks of construction in both osteoporotic mouse model, which is earlier than that of decrease of BMD. Indeed, it has been reported that estrogen deficiency expands hemopoietic stem and progenitor cells and mature blood lineages (41), and iron exerts an important role on mature white blood cell differentiation (45). Ovariectomy and iron overload may lead to the increase of peripheral white blood cells in ther early stage of both osteoporotic mouse model construction. In addition, immune cells have been shown to directly or indirectly influence bone homeostasis *via* factors including OPG/RANKL, inflammatory cytokines such as IL-6 and TNFα and other mediators secreted by immune cells (46, 47).

Osteoporotic bone microenvironment may also impact hematopoietic lineage differentiaion to maintain the elevated output of circulating blood cells. Hematopoietic stem cells residing within the specialized bone marrow niche provide continuous supply of circulating blood cells (48–50). Disruption or perturbation of bone homestasis has a profound and central role in defining the operational structure of the HSC niche (24, 51, 52). However, hematopoiesis is also finely regulated by many factors such as aging (53). With aging, a chronic low-grade inflammatory phenotype is associated with elevated white blood cell (54, 55). Consistent with this, we also found that the levels of peripheral white blood cells were mild increase in elder control mice (24-weeks-post construction), which may account for the small difference beteen OP mice and control ones at this time point. On the other hand, stessed hemotopoiesis driven by osteoporosis may also be redressed by other unclear factors in the long-term adaption, leading to the decline of circulating white blood cell counts but still high in the elder OP mice conpared to that of in control ones. The exact mechanism of how hematopoiesis was regulated in osteoporosis remains unclear and requires further investigation.

In summary, our study has demonstrated a causal link that osteoporosis is accompanied by the altered circulating blood cells, which may provide a potential diagnostic and putative marker for osteoporosis. Due to the close interplay between hematopoiesis and bone homeostasis, our study also provides insights for the pathogenesis of elevated WBC-related diseases of unknown etiology.

## Data availability statement

The original contributions presented in the study are included in the article/supplementary material. Further inquiries can be directed to the corresponding authors.

## Ethics statement

The animal study was reviewed and approved by Institutional Animal Care and Use Committees at Shandong University.

## Author contributions

YL conceived of the paper with the guidance of BZ; YL wrote the original draft; YL, QZ, ML and ZZ performed the animal experiments; JG and WH collected the clinical data; BL, SS, TYS and SD entered the clinical data; YS, TS and BZ reviewed and edited the paper. All authors have read and agreed to the published version of the manuscript.

## Funding

This work was supported by grants from Taishan Scholars Program (TSQN201812015), Rongxiang Regenerative Medicine Foundation (2019SDRX-04) and the program for Multidisciplinary Research and Innovation Team of Young Scholars of Shandong University (2020QNQT007). This work was also supported by the Postdoctoral Science Foundation of China (2021M691956), the Natural Science Foundation of Shandong Province (ZR2021QH087) and the Medicine and Health Science and Technology Development Plan Project of Shandong Province (202002040581), the Natural Science Foundation of Jiangsu Province (BK20210110) and the Science and Technology Development Project of Suzhou (SYG202119).

## Acknowledgments

We thank Shu’e Wang (Shandong University) and Xiaodong He (Shandong University) for the assistance in the detection of BMD in mice.

## Conflict of interest

The authors declare that the research was conducted in the absence of any commercial or financial relationships that could be construed as a potential conflict of interest.

## Publisher’s note

All claims expressed in this article are solely those of the authors and do not necessarily represent those of their affiliated organizations, or those of the publisher, the editors and the reviewers. Any product that may be evaluated in this article, or claim that may be made by its manufacturer, is not guaranteed or endorsed by the publisher.
